# Supplementary material for: WordCommentsAnalyzer: A windows software tool for qualitative research
Source: F1000Res. 2018 Sep 4;7:536. Originally published 2018 May 3. [Version 2] doi: 10.12688/f1000research.14819.2 (PMC6137411; doi:10.12688/f1000research.14819.2)
Supplement: Supplementary file 3 [file f1000research-7-17614-s0002.tgz › b2a79496-4ada-47c4-9682-a99ce867836d.zip!/Eight Iranian Health Journals/Journal of Health System Research _5.docx]

© Journal of Health System Research . This work is licensed under CC BY-NC 4.0 (https://creativecommons.org/licenses/by-nc/4.0/).

Volume & Issue: دوره 13، شماره 1، 1396

Title: بررسی مطالعات نگهدارنده‌های ضد میکروبی اسید بنزوئیک و بنزوات سدیم در فرآورده‌های مختلف غذایی در ایران: مروری بر شواهد موجود

http://hsr.mui.ac.ir/index.php/jhsr/article/view/2991

مقدمه: اسید بنزوئیک و بنزوات سدیم نگهدارنده‌های ضد میکروبی هستند که در فرآورده‌های غذایی کاربرد دارند. سازمان‌های ملی نظارتی، حد مجاز این دو ماده را جهت استفاده در فرآورده‌های مختلف غذایی مشخص نموده‌اند. پژوهش حاضر با مروری نظام‌مند، به بررسی میزان اسید بنزوئیک و بنزوات سدیم در مطالعات انجام گرفته در فرآورده‌های مختلف غذایی در ایران و مقایسه آن با حد مجاز مشخص شده در استاندارد ملی ایران پرداخت. روش‌ها: کلید واژه‌های Benzoic acid، Sodium benzoate، Food و Iran و معادل فارسی آن‌ها در بانک‌های اطلاعاتی ملی و بین‌المللی SID، Magiran، Google Scholar، PubMed و ISI مورد جستجو قرار گرفت. از 61 تحقیق انجام شده طی سال‌های 2000 تا 2016 میلادی، 43 مقاله حذف و 18 مقاله وارد پژوهش گردید. یافته‌ها: در تحقیقات صورت گرفته، وجود بنزوات سدیم در چهار گروه لبنیات، فرآورده‌های غذایی اسیدی، آشامیدنی‌ها و غلات بررسی شد. بیشترین مطالعات بر روی فرآورده‌های لبنی متمرکز بود و سهم کمی از پژوهش‌ها به بقیه محصولات غذایی اختصاص داشت. عدم تطابق مقدار مجاز اسید بنزوئیک و بنزوات سدیم در فرآورده‌های مختلف غذایی با استاندارد ملی ایران، در کلیه مقالات مرتبط با فرآورده‌های لبنی، اسیدی و غلات مشاهده گردید. در 80 درصد از مطالعات مربوط به فرآورده‌های آشامیدنی نیز عدم تطابق با مقادیر تعیین شده در استاندارد ملی ایران گزارش شد. نتیجه‌گیری: مطالعات صورت گرفته در ایران دو فرضیه را در خصوص اسید بنزوئیک و بنزوات سدیم در فرآورده‌های غذایی مطرح می‌نماید. فرضیه اول این که وجود اسید بنزوئیک و بنزوات سدیم در فرآورده‌های لبنی، اسیدی و غلات می‌تواند به واسطه فعل و انفعالات طبیعی ناشی از میکروارگانیسم‌ها باشد. بنابراین، انجام مطالعات جامع علمی جهت تعیین حد طبیعی تشکیل شده از اسید بنزوئیک و بنزوات سدیم در فرآورده‌های غذایی که واکنش‌های میکروبی در آن‌ها صورت می‌گیرد، نیاز است و بازنگری استانداردهای ملی مرتبط پیشنهاد می‌شود. فرضیه دوم می‌تواند مربوط به افزودن نگهدارنده‌های اسید بنزوئیک و بنزوات سدیم به فرآورده‌های غذایی توسط تولید کنندگان این گروه از محصولات باشد. بنابراین، برنامه‌ریزی و مدیریت در راستای پایش دقیق و مستمر این ترکیبات توسط سازمان‌های نظارتی و متولی سلامت توصیه می‌شود.

Title: تأثیر یخ خنک کننده در جلیقه بر روی شاخص‌های فیزیولوژیک در شرایط آب و هوایی گرم و خشک دراتاقک شرایط جوی

http://hsr.mui.ac.ir/index.php/jhsr/article/view/2317

مقدمه: کاهش بار گرمایی بدن در مدیریت استرس گرمایی از اهمیت زیادی برخوردار است. هدف از انجام مطالعه حاضر، بررسی قابلیت خنک کنندگی جلیقه حاوی بسته‌های ژل- یخ در شرایط گرم و خشک بود. روش‌ها: این مطالعه از نوع مداخله‌ای- تجربی بود و بر روی 12 دانشجوی مرد انجام گرفت. شاخص نمره استرین گرمایی (Heat Strain Score Index یا HSSI)، دمای پوست، دمای دهانی و ضربان قلب در دو حالت با و بدون استفاده از جلیقه به مدت 30 دقیقه در اتاقک شرایط جوی با میانگین دمای 8/38 درجه سانتی‌گراد و رطوبت 9/32 درصد و در دو شدت فعالیت با سرعت‌های 4/2 و 8/4 کیلومتر بر ساعت اندازه‌گیری گردید. داده‌ها با استفاده از آزمون‌های تحلیل واریانس با اندازه‌های مکرر و Independent t مورد تجزیه و تحلیل قرار گرفت. یافته‌ها: میانگین دمای پوست سینه و شاخص HSSI در در دو حالت با و بدون استفاده از جلیقه در سرعت 4/2 کیلومتر بر ساعت معنی‌دار (005/0 > P) و در سرعت 8/4 کیلومتر بر ساعت معنی‌دار نبود. همچنین، اختلاف معنی‌داری بین میانگین ضربان قلب، دمای دهانی و دمای پوست کتف در هر دو سرعت مشاهده نشد. نتیجه‌گیری: استفاده از جلیقه خنک کننده حاوی بسته‌های یخ در آب و هوای گرم و خشک، باعث کاهش میزان دمای پوست و نمره شاخص استرین گرمایی در فعالیت سبک نسبت به فعالیت متوسط می‌شود.

Title: بررسی تأثیر مداخله آموزشي مبتني بر الگوي اعتقاد بهداشتي بر ارتقای رفتارهاي پيشگيری کننده از ايدز در دختران دانش‌آموز مقطع متوسطه شهرستان مشهد در سال 1395

http://hsr.mui.ac.ir/index.php/jhsr/article/view/2899

مقدمه: بر اساس نظر سازمان جهانی بهداشت، مؤثرترین راه علیه ایدز، آموزش بهداشت است. نوجوانان دختر از جمله گروه‌های در معرض خطر در برابر ایدز می‌باشند. مطالعه حاضر با هدف تعيين اثربخشي مداخله آموزشي مبتني بر الگوي اعتقاد بهداشتي بر ارتقای رفتارهاي پيشگيري كننده از ايدز در دختران دانش‌آموز مقطع متوسطه شهرستان مشهد انجام شد. روش‌ها: در اين مطالعه نیمه تجربي، 80 دانش‌آموز دختر مقطع متوسطه شهرستان مشهد در سال 1395 در دو گروه 40 نفره آزمون و شاهد قرار گرفتند. مداخله آموزشی براي گروه آزمون به روش سخنراني و بحث گروهي انجام شد. ابزار جمع‌آوری داده‌ها، پرسش‌نامه سه قسمتي شامل مشخصات شخصی، آگاهي و سازه‌هاي مدل اعتقاد بهداشتي بود. داده‌ها با استفاده از آزمون‌های Paired t، Independent t و 2c در نرم‌افزار SPSS مورد تجزيه و تحليل قرار گرفت. یافته‌ها: اختلاف معنی‌داری میان متغیرهای دموگرافیک دو گروه مورد بررسی مشاهده نشد. قبل از مداخله اختلاف معني‌داري بین دو گروه در متغیرهای آگاهي، حساسیت، شدت، منافع، موانع درک شده و خودکارامدی وجود نداشت (050/0 < P)، اما بعد از مداخله در متغیرهای ذکر شده به جز سازه حساسیت درک شده، اختلاف معنی‌داری مشاهده شد (050/0 > P). نتیجه‌گیری: نتایج پژوهش حاضر تأثير و کارايي الگوی اعتقاد بهداشتي را در اتخاذ رفتارهاي پيشگيري کننده از ايدز تأکيد نمود و نشان داد که آموزش مبتنی بر الگوی اعتقاد بهداشتي، باعث افزایش شدت، منافع درک شده و خودکارامدی و کاهش موانع درک شده در دختران دانش‌آموز می‌شود. ایجاد حساسیت در این گروه سنی نیازمند مداخله طولانی مدت می‌باشد.

Title: تأثیر مصرف چای سبز بر میزان شاخص توده بدنی، فشار خون و نبض در کارکنان اتاق عمل

http://hsr.mui.ac.ir/index.php/jhsr/article/view/2855

مقدمه: چاقی یکی از مهم‌ترین عوامل خطر در بروز بیماری‌هاي غیر واگیر می‌باشد که حتی در کارکنان درمانی نیز شیوع رو به افزایشی دارد و می‌تواند زمینه‌ساز بیماری‌های مختلفی از جمله مشکلات قلبی- عروقی شود. روش‌های درمانی مختلفی در این زمینه ارایه شده است که یکی از آن‌ها، استفاده از چای سبز می‌باشد. بنابراین، هدف از انجام مطالعه حاضر، بررسی تأثیر چای سبز بر میزان شاخص توده بدنی (Body mass index یا BMI)، فشار خون و نبض در کارکنان اتاق عمل بود. روش‌ها: این مطالعه به صورت کارآزمایی بالینی قبل و بعد، بر روی 24 نفر از کارکنان اتاق عمل بیمارستان شهید مطهری فولادشهر اصفهان در سال 1394 انجام شد. بعد از اخذ رضایت‌نامه، از نمونه‌ها درخواست گردید تا روزانه 4 فنجان چای سبز حاوی 3 گرم برگ چای سبز در 300 میلی‌لیتر آب جوش (با دمای 80 درجه سانتی‌گراد) را به مدت 8 هفته مصرف نمایند. میزان BMI، نبض و فشار خون قبل و بعد از مصرف چای سبز اندازه‌گیری شد. در نهایت، داده‌ها در نرم‌افزار SPSS مورد تجزیه و تحلیل قرار گرفت. یافته‌ها: فشار سیستولیک و تعداد نبض بعد از 8 هفته مصرف چای سبز به طور معنی‌داری کاهش یافت، اما مصرف آن بر BMI و فشار دیاستولیک مؤثر نبود. نتیجه‌گیری: مصرف چای سبز می‌تواند جهت کاهش فشار خون سیستولیک و نبض مفید باشد و باعث بهبود وضعیت قلبی- عروقی می‌شود.

Title: تأثیر آموزش همسران بر انجام فعالیت بدنی در زنان مبتلا به سندرم پیش از قاعدگی

http://hsr.mui.ac.ir/index.php/jhsr/article/view/2443

مقدمه: سندرم قبل از قاعدگی (Premenstrual syndrome یا PMS) تركيبي ازاختلالات جسمي، عصبي و رواني است كه بر سازگاري اجتماعي، ارتباط درون فردي، فعاليت‌هاي طبيعي و كيفيت زندگي فرد تأثير منفي مي‌گذارد. فعاليت بدني یکی از شيوه‌هاي مناسب در جهت کنترل علایم این سندرم می‌باشد. با توجه به اهمیت مشارکت همسران در بهداشت باروری و ارتقای سلامت زنان، پژوهش حاضر به بررسی تأثیر آموزش همسران بر انجام فعالیت بدنی در زنان مبتلا به PMS پرداخت. روش‌ها: این مطالعه از نوع نیمه تجربی بود که در سال 1393 انجام شد. نمونه‌های مطالعه شامل 100 زوج در سن باروری مراجعه کننده به مراکز بهداشتی شهرستان فلاورجان بود که به طور تصادفی به دو گروه آزمون و شاهد تقسیم شدند. همسران گروه آزمون طی دو جلسه در زمینه فعالیت بدنی در کنترل علایم PMS آموزش دیدند. اطلاعات علایم PMS و ورزش به ترتیب با استفاده از پرسش‌نامه سنجش PMS (Premenstrual syndrome assessment یا PSA) و طرح سبا وزارت بهداشت قبل و سه ماه بعد از مداخله جمع‌آوري شد. داده‌ها در نرم‌افزار SPSS مورد تجزیه و تحلیل قرار گرفت. یافته‌ها: افزایش معنی‌داری در انجام فعالیت بدنی زنان گروه آزمون بعد از مداخله مشاهده شد (001/0 > P)، اما میزان شاخص توده بدنی (Body mass index یا BMI) کاهش معنی‌داری را نسبت به قبل و نسبت به گروه شاهد نشان نداد. علایم جسمی و روحی- رفتاری در گروه آزمون به طور معنی‌داری کمتر از گروه شاهد گزارش گردید (050/0 > P). نتیجه‌گیری: مردان به عنوان یک رابط آموزشی، به خوبی توانسته بودند زنان را به انجام فعالیت بدنی تشویق نمایند و علایم PMS را در آنان کاهش دهند. پیشنهاد می‌شود دوره‌های آموزشی ویژه مردان جهت تشویق زنان به ورزش در سیستم بهداشت و درمان اجرا گردد.

Title: ارتباط باورهای مرتبط با دارو درمانی و میزان تبعیت از درمان در بیماران مبتلا به پرفشاری خون مراجعه کننده به مراکز بهداشتی- درمانی شهر اصفهان

http://hsr.mui.ac.ir/index.php/jhsr/article/view/2555

مقدمه: تبعیت از درمان، عامل مهمی در درمان پرفشاری خون می‌باشد. برخی مطالعات نشان داده‌اند که باورهای مرتبط با دارو بر تبعیت از درمان مؤثر است. بنابراین، مطالعه حاضر با هدف تعیین ارتباط باورهای مرتبط با دارو درمانی و میزان تبعیت از درمان در بیماران مبتلا به پرفشاری خون انجام شد. روش‌ها: در این مطالعه مقطعی، 300 بیمار مبتلا به پرفشاری خون به صورت نمونه‌گیری تصادفی طبقه‌‌ای، از بین مراجعان به مراکز بهداشتی- درمانی شهر اصفهان در سال 1394 انتخاب شدند. اطلاعات با استفاده از پرسش‌نامه اطلاعات دموگرافیک، پرسش‌نامه باور‌های دارویی (Beliefs about medicines questionnaire یا BMQ) و پرسش‌نامه تبعیت از درمان (Morisky Medication Adherence Scale یا MMAS) جمع‌آوری گردید. پایایی پرسش‌نامه‌های BMQ و MMAS در مطالعات داخلی به ترتیب با 71/0 و 69/0 تأیید شده است. داده‌ها با استفاده از آزمون‌های Independent t و ضرایب همبستگی Pearson و Spearman در نرم‌افزار SPSS مورد تجزیه و تحلیل قرار گرفت. 050/0 > P به عنوان سطح معنی‌داری داده‌ها در نظر گرفته شد. یافته‌ها: میانگین سن شرکت کنندگان 16/6 ± 55/58 سال بود و 3/68 درصد از آنان را زنان تشکیل دادند. بین باورهای مرتبط با دارو درمانی و میزان تبعیت از درمان رابطه معنی‌داری مشاهده شد (21/0 = r و 001/0 = P). سن و جنسیت بیماران ارتباط معنی‌داری را با تبعیت از درمان و باورهای دارویی نشان نداد (050/0 < P). رابطه بین سطح تحصیلات و شغل با نمره باورهای دارویی معنی‌دار بود (050/0 > P)، اما ارتباط معنی‌داری بین سطح تحصیلات و شغل با تبعیت از درمان وجود نداشت (050/0 < P). نتیجه‌گیری: باورهای دارویی می‌تواند در میزان تبعیت از درمان مؤثر باشد. همچنین، مداخلات آموزشی در مبتلایان به بیماری پرفشاری خون با تأکید بر ارتقای باورهای مثبت افراد نسبت به مصرف دارو‌ها، میزان تبعیت از درمان را افزایش می‌دهد.

Title: اولویت‌بندی گزینه‌های مختلف مدیریت پسماندهای بیمارستانی در شهر تهران با استفاده از تحلیل تصمیم‌گیری چند معیاره

http://hsr.mui.ac.ir/index.php/jhsr/article/view/2761

مقدمه: در سال‌های اخیر، با توجه به افزایش مشکلات زیست ‌محیطی ناشی از پسماندهای مراکز درمانی، تعیین روش بهینه مدیریت پسماندهای بیمارستانی با در نظر گرفتن معیارهای متعدد و تا حدودی متناقض به یکی از مهم‌ترین چالش‌ها تبدیل شده است. مطالعه حاضر، با هدف ارزیابی گزینه‌های مختلف مدیریت پسماندهای بیمارستانی در شهر تهران و اولویت‌بندی آن‌ها با استفاده از تحلیل تصمیم‌گیری چند معیاره انجام شد. روش‌ها: مطالعه از نوع توصیفی- کاربردی و به لحاظ جمع‌آوري داده‎ها از نوع تحقيق پيمايشي بود که در سال 1394 انجام شد. تعداد 46 نفر از مطلعان و صاحب‌نظران این موضوع با استفاده از نمونه‌گيري هدفمند وارد مطالعه شدند. جمع‌آوری داده‌ها با استفاده از پرسش‌نامه انجام شد. روایی پرسش‌نامه طراحی شده با استفاده از نظر استادان و خبرگان، به صورت روایی صوری مورد تأیید قرار گرفت و پایایی ابزار پژوهش نیز بر اساس نرخ ناسازگاری استاندارد که کمتر از 1/0 است، با نرخ 00256/0 تأیید گردید. جهت تحلیل داده‌ها، از نرم‌افزارهای Excel و Lingo استفاده شد. یافته‌ها: در مطالعه حاضر، 12 معیار مختلف الویت‌بندی برای روش‌های دفع پسماند به دست آمد. سه معیار اثربخشی عمل، پسماندهای هوایی، تأثیرات محیطی و موانع پذیرش عمومی بیشترین وزن و سه معیار هزینه، بو و رها کردن با اثرات بهداشتی کمترین وزن را به خود اختصاص داد. در نهایت، بر اساس معیارهای فوق، روش‌های مختلف دفع پسماندهای بیمارستانی به ترتیب استريل بخار (اتوکلاو)، سوزاندن، دفن كردن، گندزدایی شیمیایی، پرتودهی و ماكروويو رتبه‌بندی شد. نتیجه‌گیری: بیشتر گروه‌های شرکت کننده در پژوهش به بحث اتوکلاو و استریل با بخار و دفن کردن بعد از خنثی‌سازی تأکید داشتند که میزان استقبال مسؤولان و دست‌اندرکاران از روش‌های حداقل تأثیر بر محیط زیست را نشان می‌دهد. با این حال، جنبه‌های هزینه‌ای و مالی روش‌های مختلف دفع پسماندهای بیمارستانی نیز باید مد نظر قرار گیرد.

Title: بررسي شاخص‌های کیفی آب استخرهای شنا در شاهین‌شهر در سال 1390

http://hsr.mui.ac.ir/index.php/jhsr/article/view/2797

مقدمه: شنا یکی از ورزش‌های مهم برای سلامت جسم و روح است که عدم رعایت موازین بهداشتی در استخرها باعث بروز مشکلات بهداشتی و انتقال بیماری‌های عفونی به شناگران می‌شود. هدف از انجام مطالعه حاضر، بررسی شاخص‌های کیفی آب استخرهای شنای شاهین‌شهر و مقایسه آن با استاندارد بود. روش‌ها: مطالعه حاضر توصیفی- تحلیلی بود که به صورت مقطعی طی سال‌های 91-1390 انجام گرفت. از 3 استخر فعال شهرستان شاهین‌شهر، 288 نمونه آب در مدت 4 ماه به صورت سه هفته یک بار تهیه گردید. پارامترهای کلر آزاد باقیمانده، کدورت، pH، دما و همچنین، پارامترهای میکروبی نظیر باکتری هتروتروف، سودوموناس آئروژینوزا، کلیفرم کل و مدفوعی مورد آنالیز قرار گرفت. یافته‌ها: میزان pH در 88/13، کلر آزاد باقیمانده در 11/36، کدورت در 77/52 و دما در 22/22 درصد نمونه‌ها مطلوب بود. جمعیت باکتری‌های هتروتروف، سودوموناس آئروژینوزا و کلیفرم کل و مدفوعی به ترتیب در 22/72، 11/86، 88/88 و 88/88 درصد موارد مطلوب به دست آمد. در آنالیز آماری داده‌ها، بین کلیفرم مدفوعی و کدورت رابطه مستقیم وجود داشت. نتیجه‌گیری: بر اساس نتايج، پارامترهاي pH، دما و کلر باقیمانده كمترين تطابق را با استاندارد داشت كه نشان دهنده ضرورت پايش مداوم پارامترهاي فيزيكي و شيميايي آب و همچنين، گندزدايي آب استخر مي‌باشد.

Title: نقش مؤلفه‌های حمایت اجتماعی در سلامت عمومی سالمندان

http://hsr.mui.ac.ir/index.php/jhsr/article/view/2808

مقدمه: یکی از مراحل اساسی زندگی بشر، دوران سالمندی است که همراه با نیازها، خواسته‌ها و مسایل متفاوت می‌باشد. تحقیق حاضر، با هدف تعیین نقش حمایت اجتماعی در سلامت عمومی سالمندان انجام شد. روش‌ها: روش مورد استفاده در پژوهش، پیمایش و ابزار جمع‌آوری اطلاعات پرسش‌نامه بود. جامعه آماری، کلیه شهروندان سالمند بالای 65 سال و حجم نمونه 265 نفر انتخاب شد. روش نمونه‌گیری ترکیبی از طبقه‌ای و خوشه‌ای چند مرحله‌ای بود که داده‌ها از 9 محله منتخب در شهر مشهد در سال 1394 جمع‌آوری شد. همچنین، جهت تجزیه و تحلیل داده‌ها از آزمون رگرسیون و آزمون مقایسه میانگین t مستقل استفاده گردید. یافته‌ها: سلامت عمومی سالمندان در حد متوسط و بالاترین میزان سلامت عمومی به خرده مقیاس سلامت اجتماعی تعلق داشت. همچنین، حمایت اجتماعی از سالمندان در حد متوسط و بیشترین میزان حمایت متعلق به خرده مقیاس عاطفی گزارش شد. نتایج آزمون t مستقل نشان داد که سلامت عمومی مردان به طور معنی‌داری بالاتر از زنان بود. در نهایت، در بین ابعاد حمایت اجتماعی، بعد ‌عاطفی بیشترین تأثیر را بر سلامت عمومی سالمندان داشت. نتیجه‌گیری: حمايت عاطفي بيشترين تأثير را بر سلامت عمومی سالمندان داشت، اما بايد توجه کرد که سالمندان در کنار نیازهای عاطفی، به حمايت‌هاي خدماتي زیاد، مالي و مشورتي هم احتياج دارند.

Title: بررسی سطح سرمی عنصر روی و رابطه آن با کارکرد شناختی و تحصیلی در دانش‌آموزان دختر هنرستان‌های شهرستان ایذه

http://hsr.mui.ac.ir/index.php/jhsr/article/view/2860

مقدمه: تغذیه یکی از مهم‌ترین عوامل محیطی تأثیرگذار بر کارکرد شناختی محسوب می‌شود. روی از عناصر ریزمغذی است که کمبود آن در ابتدای زندگی عوارض جبران‌ناپذیر و در مراحل بعدی رشد، عوارض شناختی قابل برگشتی بر جای می‌گذارد. هدف از انجام مطالعه حاضر، بررسی سطح سرمی عنصر روی و ارتباط آن با کارکرد شناختی و تحصیلی دانش‌آموزان دختر هنرستانی بود. روش‌ها: در این پژوهش مقطعی، 100 دانش‌آموز دختر هنرستانی انتخاب و سطح سرمی عنصر روی با استفاده از روش جذب اتمی اندازه‌گیری شد. کارکرد شناختی شامل بهره هوشی، حافظه و تمرکز با استفاده از آزمون‌های بهره هوشی Raven، آزمون حافظه Wechsler (Wechsler Memory Scale یا WMS) و آزمون‌های خط‌زنی (آزمون دقت Bonardel و خط‌زنی حروف) مورد بررسی قرار گرفت. وضعیت تحصیلی نیز با استفاده از معدل تحصیلی موجود در پرونده تحصیلی محاسبه گردید. از رگرسیون تعمیم یافته (Generalized linear model یا GLM) برای بررسی تأثیر سطح سرمی عنصر روی بر تغییرات میزان نمرات آزمون‌های بهره هوشی، حافظه، تمرکز و کارکرد تحصیلی استفاده شد. یافته‌ها: میانگین سطح سرمی عنصر روی، 00/31 ± 51/105 میکروگرم بر دسی‌لیتر بود. میانگین نمرات آزمون‌های بهره هوشی Raven، WMS، دقت Bonardel، خط‌زنی حروف و معدل تحصیلی به ترتیب 80/11 ± 44/91، 2/11 ± 6/84، 6/0 ± 0/46، 20/5 ± 84/44 و 80/1 ± 51/14 به دست آمد. بین سطح سرمی عنصر روی و آزمون‌های بهره هوشی Raven، WMS، خط‌زنی حروف و حذف عدد رابطه معنی‌داری وجود داشت (050/0 > P)، اما بین سطح سرمی روی و معدل تحصیلی ارتباطی مشاهده نشد. نتیجه‌گیری: بین کارکرد شناختی از جمله بهره هوشی، حافظه و تمرکز با سطح سرمی عنصر روی رابطه وجود دارد. لازم است نسبت به آموزش مصرف مواد غذایی حاوی این عنصر جهت پیشرفت تحصیلی توجه شود.

Title: کارایی خاکستر ریشه گیاه شیرین بیان در حذف رنگ

http://hsr.mui.ac.ir/index.php/jhsr/article/view/2458

مقدمه: رنگ یکی از مهم‌ترین آلاینده‌های موجود در فاضلاب نساجی به شمار می‌رود که اغلب سمی، سرطان‌زا و جهش‌زا می‌باشد. بنابراین، آلاینده‌های رنگی قبل از ورود به محیط زیست باید تصفیه شوند. مطالعه حاضر با هدف بررسی کارایی خاکستر ریشه گیاه شیرین بیان در حذف رنگ راکتیو بلک 5 از محلول‌های آبی انجام شد. روش‌ها: این مطالعه به صورت تجربی و در مقیاس آزمایشگاهی انجام گرفت. جاذب مورد نظر در شرایط آزمایشگاهی تهیه و با استفاده از الک‌های استاندارد دانه‌بندی شد و اثر شاخص‌های مختلف شامل pH (12-2)، غلظت اولیه رنگ (60-40-20 میلی‌گرم در لیتر)، زمان تماس (180-10 دقیقه) و دوز جاذب (2-3/0 گرم در 100 سی‌سی) مورد بررسی قرار گرفت. یافته‌ها: کارایی خاکستر ریشه گیاه شیرین بیان در حذف رنگ با افزایش pH و غلظت اولیه رنگ، کاهش و با افزایش زمان تماس تا 60 دقیقه اول افزایش یافت. همچنین، یافته‌ها از ایزوترم Langmuir (9467/0 = 2R) بهتر پیروی نمود. نتیجه‌گیری: خاکستر ریشه گیاه شیرین بیان از توانایی مطلوبی (48/98 درصد در غلظت 20 میلی‌گرم آلاینده) در حذف رنگ راکتیو بلک 5 از محلول‌های آبی و فاضلاب‌های صنعت نساجی برخوردار است.

Title: تغييرات آلاينده‌هاي خروجي از دودکش بخاري گاز‌سوز خانگي در طي ماه‌هاي سرد، 92-1391

http://hsr.mui.ac.ir/index.php/jhsr/article/view/2464

مقدمه: مصرف عمده گاز طبیعی در ايران، استفاده از آن به عنوان منبع گرمایشی است که بيشتر از طریق احتراق در بخاری‌های گازی ایجاد می‌شود. بخاری‌های خانگی به علت تعداد زياد، پتانسیل تأثیر قابل توجهی در تولید گازهای گلخانه‌ای و آلاینده دارند. بنابراين، هدف از انجام تحقیق حاضر، بررسی ميزان انتشار آلاینده‌های گازی خروجی از دودکش بخاری گازسوز خانگی و ارتباط آن با شرایط دمای جوی و مصرف کلی گاز در کشور بود. روش‌ها: در اين مطالعه، جهت سنجش آلاینده‌های گازی یک بخاری گازسوز متداول خانگی، از دستگاه سنجش آلاینده‌های خروجی از دودکش، مدل Lancom III استفاده گردید. تحقیق طی پنج ماه سرد در سال‌های 1391 تا 1392 انجام شد. داده‌های دمایی هوا از اداره هواشناسی و میزان کلی مصرفی گاز از شرکت ملی گاز به دست آمد. یافته‌ها: مقدار میانگین خروجی گازهای 2CO، 2SO، 2NO، ‏NO‎‏ و ‏CXHY‏ به ترتیب 64/10939، 091/0، 01/1، 99/6 و 996/172 ppm بود. ارتباط معني‌داري بين کاهش دماي روزانه و افزايش میزان 2SO و CXHY وجود داشت (010/0 > P). همچنین، ارتباط معني‌داري میان کاهش دماي روزانه و کاهش 2CO و ‏2NO ‎‏در خروجي دودکش مشاهده شد (050/0 > P). نتیجه‌گیری: افزایش غلظت ‏2SO و CXHY و کاهش غلظت 2CO و ‏2NO در روزهای سرد، مي‌تواند به علت تغییرات در کیفیت گاز مصرفی باشد. در چنین روزهايی مصرف کلي گاز در کشور افزايش مي‌يابد و بنابراین، باعث تغییراتی در دبي، سرعت و فشار گاز در لوله‌های انتقال و نیز کيفيت گاز (انتقال نشست‌هاي گوگردي از لوله‌های به جريان گاز) مي‌شود.

Title: بررسی رابطه بین مشارکت ورزشی با کیفیت زندگی زنان شهر اصفهان

http://hsr.mui.ac.ir/index.php/jhsr/article/view/2823

مقدمه: امروزه یکی از ابزارهای سهل‌الوصول، ارزان و تعیین کننده سلامتی انسان‌ها، فعالیت‌های حرکتی و ورزش است و از سوی دیگر، کیفیت زندگی یکی از پیامد‌های مهم سلامتی به شمار می‌آید. بنابراین، هدف از انجام تحقیق حاضر، تعیین رابطه بین مشارکت ورزشی با کیفیت زندگی زنان شهر اصفهان بود. روش‌ها: این پژوهش از نوع پیمایشی بود و به روش توصیفی- همبستگی در سال 1394 انجام شد. جامعه آماری مطالعه را کلیه زنان شهر اصفهان (919399 نفر) تشکیل دادند. از این تعداد، 386 نفر به روش تصادفی طبقه‏ای و با استفاده از جدول تعیین حجم نمونه Morgan انتخاب شدند. جهت جمع‌آوری داده‌ها، از پرسش‌نامه استاندارد کیفیت زندگی (World Health Organization Quality of Life یا WHOQOL-BREF) که پایایی آن 92/0 به دست آمده و پرسش‌نامه مشارکت ورزشی کریمیان و همکاران که پایایی آن 88/0 محاسبه شده است، استفاده گردید. یافته‌ها: بین مشارکت ورزشی با کیفیت زندگی زنان، همبستگی مثبت و معنی‌داری مشاهده شد (371/0 = r، 001/0 = P). همچنین، میزان مشارکت ورزشی زنان با میانگین 03/3 و کیفیت زندگی با میانگین 22/3، در سطح متوسطی قرار داشت. با توجه به تحلیل‏های رگرسیونی، 29 درصد از کیفیت زندگی می‏تواند به وسیله مشارکت ورزشی پیش‌بینی شود. نتیجه‌گیری: ورزش و فعالیت‌های بدنی منظم، ارتباط معنی‌داری با سطح کیفیت زندگی در زنان دارد و می‌توان چنین استنباط نمود که با افزایش مشارکت ورزشی، کیفیت زندگی زنان نیز افزایش و بهبود می‌یابد.

Title: بررسی رابطه بین نوبت‌کاری با خطر ابتلا به بیماری‌های قلبی- عروقی بر اساس نمره Framingham با استفاده از مدل آمیخته تصادفی چند سطحی

http://hsr.mui.ac.ir/index.php/jhsr/article/view/2902

مقدمه: تشخیص مدل مناسب برای توزیع صفت جامعه مورد بررسی در بسیاری از مسایل کاربردی اهمیت ویژه‌ای دارد. در مطالعه حاضر سعی گردید تا با ترکیب توزیع‌های آمیخته به عنوان یکی از روش‌های داده‌کاوی و تحلیل چند سطحی به‌ عنوان روشی کارا در تحلیل داده‌های آشیانه‌ای، با رویکرد Bayesian به بررسی رابطه نوبت‌کاری و نمره خطر Framingham پرداخته شود. روش‌ها: مدل ساخته شده با هدف بررسی رابطه نوبت‌کاری و نمره Framingham، بر روی داده‌های یک مطالعه واقعی که به صورت مقطعی در شش ماهه اول سال 1394 بر روی 3243 نفر از کارکنان کارخانه فولاد مبارکه اصفهان انجام شده بود، مورد استفاده قرار گرفت. یافته‌ها: مشارکت کنندگان مطالعه شامل 1690 نفر کارگر روزکار با میانگین سنی 13/6 ± 13/42 سال، 1356 نفر نوبت‌کار گردش معمولی با میانگین سنی 09/6 ± 75/40 سال و 197 نفر نوبت‌کار هفتگی با میانگین سنی 10/6 ± 60/41 سال بود. در مطالعه حاضر با کنترل عوامل مخدوشگر، نوبت‌کاري با نمره Framingham رابطه‌ معني‌داری را نشان داد. نتیجه‌گیری: نتایج به دست آمده از مدل‌سازی حاضر، حاکی از بیشتر بودن نمره Framingham در میان نوبت‌کاران نسبت به روزکاران می‌باشد که این نتیجه با مطالعات گذشته همخوانی دارد.

Title: بررسی ارتباط وضعیت ارگونوميک مشاغل ساختمان‌سازی با استفاده از چک‌ليست NIOSH-CPWR و اختلالات اسکلتی- عضلانی در میان کارگران ساختمانی شهر اهواز

http://hsr.mui.ac.ir/index.php/jhsr/article/view/2642

مقدمه: کارگران ساختمانی رتبه دوم بیشترین آسیب‌های ناشی از اختلالات اسکلتی- عضلانی را به خود اختصاص می‌دهند. هدف از انجام مطالعه حاضر، ارزيابی وضعيت ارگونوميک مشاغل ساختمان‌سازی با استفاده از ‌چک‌لیست National Institute for Occupational Safety and Health-Center to Protect Workers Rights (NIOSH-CPWR) و پرسش‌نامه Nordic بود. روش‌ها: این مطالعه به صورت توصیفی- تحلیلی- مقطعی بر روی 106 کارگر ساختمان‌سازی شهر اهواز انجام گرفت. ابزار پژوهش، پرسش‌نامه اختصاصی Nordic و چک‌لیست ارگونومیکی ساختمان NIOSH-CPWR مشتمل بر 11 بخش اختصاصی بود. پرسش‌نامه Nordic برای 106 کارگر ساختمان‌سازی تکمیل گردید. در نهایت، داده‌ها در نرم‌افزار SPSS مورد تجزیه و تحلیل قرار گرفت. یافته‌ها: اختلال شانه (3/61 درصد)، کمر (4/59 درصد) و زانو (7/54 درصد) به ترتیب بیشترین درصد فراوانی اختلالات اسکلتی- عضلانی را در میان نمونه‌ها به خود اختصاص داد. 5/74 درصد از مشاغل حرکات تکراری داشتند و 9/68 درصد مجبور بودند بیش از یک ساعت در روز بر روی زانوها کار کنند. 5/75 درصد از کارگران مجبور به خم شدن و چرخش‌های بدنی حین کار بودند. اغلب سطوح کارگاه‌های ساختمانی نیز برای راه رفتن و کار کردن ناصاف و سخت می‌باشد. نتیجه‌گیری: اختلالات اسکلتی- عضلانی در میان کارگران ساختمانی مدت کوتاهی پس از شروع به کار آن‌ها بروز می‌کند. بنابراین، استفاده از ابزارها و تجهیزات دارای طراحی مطابق با اصول ارگونومی، کنترل‌های مدیریتی و آموزش دستورالعمل‌ها و رعایت استاندارهای مربوط از سوی مدیران و دستگاه‌های ناظر بر فعالیت‌های ساختمانی و سلامت کارگران، از جمله ضروریات برنامه‌های پایش سلامت محیط کار می‌باشد.

Title: بهینه‌سازی فرایند کنترل صدای سایلنسری از جنس پلی‌استایرن (یونولیت) و بررسی نقش آن در تغییر شاخص افت ورودی صدای منابع هنگام استفاده به صورت حصار در محیط آزمایشگاهی

http://hsr.mui.ac.ir/index.php/jhsr/article/view/2657

مقدمه: مواجهه با صداي بيش از حد مجاز، يكي از معضلات بهداشتي كارگران در دنيا محسوب مي‌گردد. یکی از مهم‌ترین راه‌های کنترل صدا، استفاده از مواد جاذب به صورت سایلنسر اطراف منبع صوت می‌باشد. بنابراین، پژوهش حاضر با هدف تعیین میزان افت ورودی صدای سایلنسری از جنس یونولیت و تغییرات ساختاری آن به منظور بهبود کاهش صوت انجام شد. روش‌ها: جهت ساخت سایلنسر، از فوم پلی‌استایرن (یونولیت) به صورت مکعب مستطیل (بدون وجه کف) با دانسیته 20 کیلوگرم بر مترمکعب و ضخامت دیواره 5 سانتی‌متر و ابعاد کلی 5/1 × 1 × 1 متر استفاده شد. همچنین، یک بلندگو به عنوان منبع تولید صدا با تراز صوت 100 دسی‌بل جهت پخش صوت به صورت عمود بر زمین و دو میکروفن نیز به منظور جمع‌آوری صدا مورد استفاده قرار گرفت. سپس با اعمال تغییراتی مانند افزایش ضخامت و ایجاد حفره روی دیواره‌ها، میزان کاهش صوت بررسی گردید. یافته‌ها: وجود سایلنسر یونولیت با ضخامت 5 سانتی‌متر و بدون حفره، سبب کاهش صوت حداکثر 5/34 دسی‌بل در فرکانس 500 هرتز شد. افزایش ضخامت به 7 سانتی‌متر و اضافه نمودن حفره به قطر 2 سانتی‌متر در فرکانس‌‌های 250، 500، 1000 و 2000 هرتز، منجر به کاهش صوت بیشتری نسبت به سایلنسر بدون تغییرات گردید. نتیجه‌گیری: به منظور افزایش بهره‌وری سایلنسری از جنس پلی‌استایرن، با توجه به شرایط اقتصادی و محل مورد نظر، می‌توان از ضخامت بیشتر این ماده استفاده نمود. همچنین، طراحی صحیح حفره بر روی دیواره‌های سایلنسر، سبب افزایش افت صدای ناشی از منبع به محیط بیرون از سایلنسر می‌گردد.

Title: بررسی الگوی اسیدهای چرب شیر خام، پاستوریزه و استرلیزه در 4 کارخانه تولید کننده فراورده لبنی در استان اصفهان و مرکزی

http://hsr.mui.ac.ir/index.php/jhsr/article/view/2842

مقدمه: مطالعه حاضر با هدف مقایسه الگوی اسیدهای چرب شیر خام و تغییرات آن‌ها طی فرآوری حرارتی، در طول سه ماه در تابستان سال 1394 انجام شد. روش‌ها: در این مطالعه مقطعی 72 نمونه شیر از صنایع تولید شیر 4 شرکت تولید شیر و فرآورده‌های لبنی در 2 استان اصفهان و مرکزی به طور تصادفی از روی خط تولید شیر پاستوریزه، استرلیزه و تانک ذخیره شیر خام طی 3 نوبت در فصل تابستان جمع‌آوری گردید. الگوی اسیدهای چرب شیر در شیر خام و حرارت دیده به روش Gas chromatography (GC) مقایسه شد. تحلیل داده‌ها به کمک آزمون‌های آماری آنالیز واریانس دو طرفه و آزمون تعقیبی least significant difference (LSD) صورت گرفت. یافته‌ها: غلظت اسیدهای چرب اشباع و غیر اشباع در شیر خام پس از فرآوری حرارتی به طور قابل توجهی کاهش یافت (05/0 > P)؛ به طوری که کاهش (60-23 درصدی) غلظت اسیدهای چرب در شیر استرلیزه نسبت به شیر خام و کاهش (74-45 درصدی) غلظت اسیدهای چرب در شیر پاستوریزه نسبت به شیر خام مشاهده گردید. علاوه بر این، نتایج نشان داد که در طول دوره سه ماهه مطالعه در فصل تابستان اثر زمان نمونه‌برداری بر غلظت اکثر اسیدهای چرب تأثیرگذار بود (05/0 > P). نتیجه‌گیری: فرایندهای حرارتی رایج در صنایع شیر موجب تغییرات اساسی در غلظت اسیدهای چرب شیر می‌شود و با وجود ملایم‌تر بودن فرایند پاستوریزاسیون شیر نسبت به فرایند استرلیزه میزان کاهش و تخریب اسیدهای چرب بیشتر مشاهده شد. از این‌رو، با توجه به اهمیت شیر پاستوریزه در سبد غذایی هر ایرانی در نظر گرفتن تغییرات فوق در جدول ترکیبات مواد غذایی حایز اهمیت است.

Title: فعالیت فیزیکی و عوامل پیش‌بینی کننده آن بر اساس مدل ارتقای سلامت Pender در دانش‌آموزان امیدیه

http://hsr.mui.ac.ir/index.php/jhsr/article/view/2181

مقدمه: انجام فعالیت فیزیکی منظم در حفظ و ارتقای سلامت جامعه تاثیر زیادی دارد. فعالیت فیزیکی منظم در بین دختران نوجوان کاهش یافته است و دلایل این امر به طور کامل مشخص نیست. مطالعه حاضر، با هدف مطالعه رفتار فعالیت فیزیکی دانش‌آموزان شهرستان امیدیه و عوامل پیش‌بینی کننده آن بر اساس مدل ارتقای سلامت Pender طراحی شد. روش‌ها: مطالعه به صورت مقطعی- تحلیلی بر روی 430 دانش‌آموز دختر دبیرستانی با روش نمونه‌گیری خوشه‌ای- تصادفی انجام گرفت. جمع‌آوری داده‌ها با کمک پرسش‌نامه‌ و تحلیل داده‌ها با آزمون‌های همبستگی، آنالیز واریانس و رگرسیون خطی صورت گرفت. یافته‌ها: از کل نمونه‌ها 25 درصد در طول هفته گذشته هیچ فعالیت فیزیکی نداشتند‌ و فقط 4/4 درصد بین 7-6 روز در هفته به مدت 30 دقیقه فعالیت فیزیکی انجام دادند. نمرات اکثر سازه‌های مدل Pender در حد میانگین نمره قابل کسب بود، اما نمره‌ برنامه‌ریزی برای عمل و خودکارامدی در نیمی از نمونه‌ها پایین‌تر از میانگین نمره‌ قابل اکتساب بود. سازه‌های مدل Pender با رفتار فعالیت فیزیکی رابطه‌ معنی‌دار داشت (05/0 > P). در کل 24 درصد از تغییرات رفتار فعالیت فیزیکی با کمک سازه‌های مدل Pender قابل پیش‌بینی بود (05/0 > P). نتیجه‌گیری: رفتار فعالیت فیزیکی دختران نوجوان در سطح قابل قبولی قرار ندارد. این نکته می‌تواند زنگ خطری برای افزایش بیماری‌های مزمن در آینده‌ این جمعیت باشد و با توجه به این که اکثر سازه‌های الگوی ارتقای سلامت Pender با رفتار فعالیت فیزیکی مرتبط بوده‌، این امر می‌تواند دستمایه‌ای برای طراحی مداخلات مؤثر جهت افزایش این رفتار در نوجوانان باشد.

Title: حذف آنیون نیترات از آب با استفاده از زئولیت کربنات کانکرینایت اصلاح شده با ماده فعال سطحی کاتیونی

http://hsr.mui.ac.ir/index.php/jhsr/article/view/2708

مقدمه: غلظت بالای آنیون نیترات در آب آشامیدنی، منجر به بروز مشکلات فراوانی برای سلامت انسان‌ها می‌شود. هدف از انجام پژوهش حاضر، سنتز و مشخصه‌یابی زئولیت کربنات کانکرینایت با خلوص بالا و سپس اصلاح سطح آن به منظور کاهش غلظت آنیون نیترات از آب بود. روش‌ها: زئولیت کربنات کانکرینایت به روش هیدروترمال و با استفاده از ماده اولیه کائولن سنتز گردید و زمان سنتز آن با پیش‌اقامت در حمام فراصوت کاهش یافت. پس از مشخصه‌یابی زئولیت سنتز شده، اصلاح سطح آن با استفاده از ماده فعال سطحی کاتیونی انجام گرفت و زئولیت اصلاح شده بهینه برای حذف نیترات از آب مورد استفاده قرار گرفت. یافته‌ها: زئولیت کربنات کانکرینایت با استفاده از ماده اولیه کائولن، غلظت سود 6 مولار و کربنات سدیم 05/0 مولار در دمای 200 درجه سانتی‌گراد با خلوص بالا سنتز گردید. زمان سنتز این زئولیت با استفاده از 90 دقیقه پیش‌اقامت در حمام فراصوت، به کمتر از نصف کاهش یافت. ظرفیت تبادل کاتیون خارجی (External Cation Exchange Capacity یا ECEC) بسیار بالای این زئولیت، اصلاح سطح آن به منظور حذف آنیون‌ها از آب را توجیه می‌کند. بر مبنای مدل Langmuir، حداکثر ظرفیت جذب نیترات روی جاذب سنتزی در دماهای 20، 30 و 40 درجه سانتی‌گراد به ترتیب 33/35، 41/28 و 06/17 میلی‌گرم بر گرم به دست آمد. نتیجه‌گیری: زئولیت کربنات کانکرینایت با خلوص و ECEC بالا، با استفاده از کائولن و با پیش‌اقامت در حمام فراصوت در زمان کوتاه قابل سنتز می‌باشد و در صورت اصلاح سطح، نسبت به زئولیت‌های طبیعی و مصنوعی دیگر، به طور مؤثرتری برای حذف آنیون نیترات از آب قابل استفاده است.

Title: حذف آرسنیک سه ظرفیتی و کروم شش ظرفیتی از محلول‌های آبی با استفاده از نانو ذرات آهن صفر ظرفیتی (nZVI) و تعیین ایزوترم‌های جذب

http://hsr.mui.ac.ir/index.php/jhsr/article/view/2662

مقدمه: آرسنیک و کروم از جمله فلزات سنگین است که به عنوان آلاینده‌های متداول منابع آب و فاضلاب مطرح می‌باشد. نانو ذرات آهن با ظرفیت صفر (Nano zero valent iron یا nZVI) به دلیل ظرفیت بالای خود قادر به حذف این آلاینده‌ها می‌باشد. هدف از انجام مطالعه حاضر، بررسی حذف آرسنیک (III) و کروم (VI) از محلول‌های آبی با استفاده از nZVI و تعیین ایزوترم‌های جذب بود. روش‌ها: جهت تعیین و تأثیر پارامترهایی مانند pH، غلظت اولیه، مقدار nZVI و زمان بر حذف آرسنیک و کروم از نرم‌افزار طراحی آزمایش استفاده شد. غلظت نهایی فلزات توسط دستگاه پلاسمای القایی جفت الکترونی (ICP یا Inductively coupled plasma) اندازه‌گیری شد. سپس، بهترین ایزوترم‌های جذب تعیین گردید. یافته‌ها: نتایج نشان دهنده حذف بهینه آرسنیک در غلظت اولیه 10 میلی‌گرم بر لیتر، زمان 5 دقیقه، 7 = pH و مقدار nZVI برابر 5 گرم بر لیتر بود. حذف آرسنیک توسط nZVI در غلظت‌های مختلف از ایزوترم‌های جذب Langmuir پیروی کرد. واکنش آرسنیک در حضور nZVI در محیط خنثی و در حضور غلظت بالای nZVI بسیار سریع رخ داد. کارایی حذف آرسنیک با افزایش غلظت رابطه عکس داشت. کارایی حذف کروم توسط nZVI بسیار پایین بود. نتیجه‌گیری: نتایج نشان داد که nZVI در حذف آرسنیک از محلول‌های آبی دارای کارایی بالایی است. بنابراین، می‌توان از آن در حذف این فلز از آب‌های آلوده و فاضلاب‌های صنعتی استفاده نمود، اما کارایی حذف کروم توسط nZVI بسیار کم می‌باشد.
